# Supplementary material for: Dichotomy of cellular inhibition by small-molecule inhibitors revealed by single-cell analysis
Source: Nat Commun. 2016 Sep 30;7:12428. doi: 10.1038/ncomms12428 (PMC5056434; doi:10.1038/ncomms12428)
Supplement: Supplementary Information — Supplementary Notes 1-4, Supplementary References [file ncomms12428-s1.pdf]

Supplementary Information

Supplementary Note 1 JAK-STAT model

Our mechanistic understanding of JAK - STAT inhibition is motivated by the biochemistry of small molecule chemical inhibitors. In the following sections we present the three mechanisms of inhibition that we tested; noncompetitive (Supplementary Figure 2), uncompetitive (Supplementary Figure 4), and competitive inhibition (Supplementary Figure 6) [1, 2].

Supplementary Note 1.1 Steady-state approximation for JAK-STAT kinetics

In the main text, and the proceeding models of pSTAT5 inhibition, we invoke a steady state approximation for our quantitative analysis. Based on experimental considerations delineated here, we chose the 15 minute time point, Supplementary Figure 1, as maximally suited for this approximation for two reasons. First, there is a minimal difference in the average abundance of pSTAT5 between the 15 minute and 30 minute time points for drug doses spanning the concentrations presented in the main text. Secondly, a 15-min timespan is sufficiently short an amount of time that complex gene regulation should not be taken under consideration. There exist additional responses to JAK inhibition (e.g. block of cell proliferation or induction of apoptosis) that may alter the signaling patterns of the cells, but these occur on much longer timescales (e.g. multiple hours) and can safely be excluded from our analysis of signaling responses.

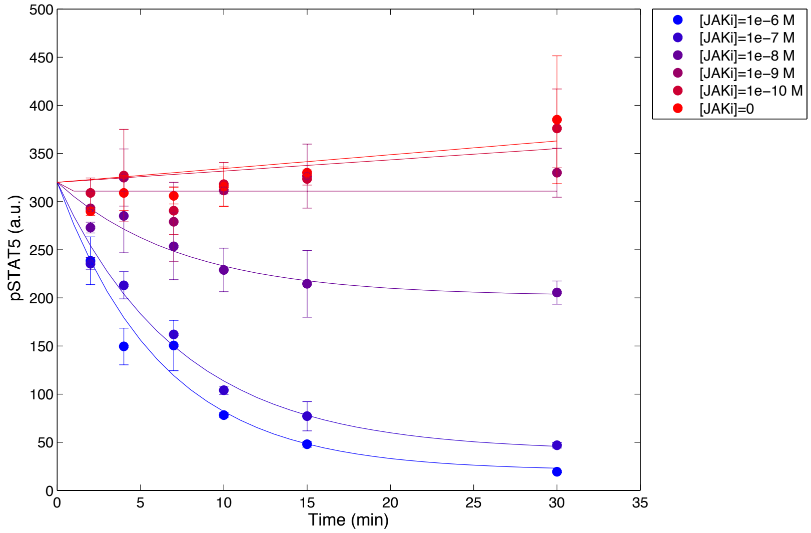

Supplementary Figure 1: **pSTAT5 Inhibition Dynamics** Measurements of pSTAT5 response of T lymphocytes to Interleukin 2 (IL-2) stimulation followed by JAK inhibition (AZD1480).

Supplementary Note 1.2 Noncompetitive inhibition

Noncompetitive inhibitors bind to their target enzyme irrespective of the presence of the enzymes substrate. Here, we consider the enzyme substrate to be the measurable abundance of the STAT5 protein. Although, while fluctuations on the ATP levels could potentially affect the enzymatic activities of the kinases under consideraion, we expect ATP levels to be maintained at homeostasis very tightly, such that we could safely assume, without lack of generality, that ATP levels were fixed in our biological system.

To develop a quantitative description of noncompetitive inhibition in living cells we start by considering

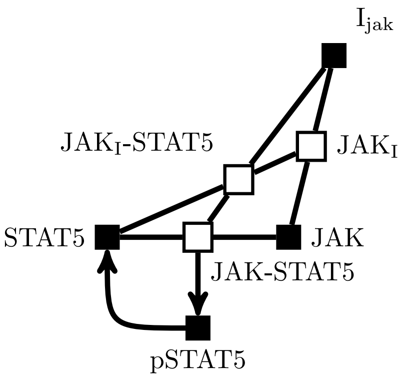

Supplementary Figure 2: Graphical representation of the noncompetitive JAK inhibitor model. The square symbols represent either a chemical species (filled) or a chemical complex (open). The interaction between chemical species and complexes is either an equilibrium reaction (lines) or unidirectional catalyzed reaction (arrows).

the set of mass action chemical kinetic equations governing the system:

$$\frac{d}{dt}[\text{pSTAT5}] = \Gamma[\text{JAK-STAT5}] - \lambda[\text{pSTAT5}], \tag{1a}$$

$$\frac{d}{dt}[\text{JAK}_\text{I}\text{-STAT5}] = k_+^{\text{Ij}}[\text{I}_\text{jak}][\text{JAK-STAT5}] - k_-^{\text{Ij}}[\text{JAK}_\text{I}\text{-STAT5}] + k_+[\text{JAK}_\text{I}][\text{STAT5}] - k_-[\text{JAK}_\text{I}\text{-STAT5}], \tag{1b}$$

$$\frac{d}{dt}[\text{JAK-STAT5}] = k_+[\text{JAK}][\text{STAT5}] - k_-[\text{JAK-STAT5}] - \Gamma[\text{JAK-STAT5}] + k_-^{\text{Ij}}[\text{JAK}_\text{I}\text{-STAT5}] - k_+^{\text{Ij}}[\text{I}_\text{jak}][\text{JAK-STAT5}]. \tag{1c}$$

Where we identify the equilibrium reaction parameters as k_+ for the association of JAK and STAT5, k_- represents the dissociation of the JAK-STAT5 complex, and $k_\pm^{\text{Ij}}$ represents the inhibitor forward and reverse rate constants. The enzymatic reactions of the phosphorylation and dephosphorylation are parameterized by rate constants Γ and λ, respectively. Conservation of mass dictates that,

$$[\text{STAT5}_\text{total}] = [\text{pSTAT5}] + [\text{STAT5}] + [\text{JAK-STAT5}] + [\text{JAK}_\text{I}\text{-STAT5}], \tag{2a}$$

$$[\text{JAK}_\text{total}] = [\text{JAK}] + [\text{JAK}_\text{I}] + [\text{JAK-STAT5}] + [\text{JAK}_\text{I}\text{-STAT5}]. \tag{2b}$$

However, we simplify the STAT5 constraint by considering the relative abundance of both JAK and STAT5 in our experimental system. Feinerman *et al* [3] previously presented values of the abundance of $[\text{STAT5}_\text{total}]$ that are nearly an order of magnitude greater than the IL2 Receptor β chain. The IL2Rβ chain is important for it is the limiting component of the active IL2 Receptor, JAK1, and JAK3 complex [3]. As a result, the total effective abundance of active JAK in our system ($[\text{JAK}_\text{total}]$) is negligible when considered with the STAT5 abundance - motivating our approximation of $[\text{STAT5}_\text{total}] \approx [\text{pSTAT5}] + [\text{STAT5}]$.

In addition we simplify the dynamic equations by using a quasi-steady state approximation, which assumes that the binding / debinding kinetics of the equilibrium reactions are much faster than the catalytic reactions. This separation of time-scales between the equilibrium and the catalytic reactions simplifies the dynamics by setting all the time derivatives of the equilibrium reactions to zero. Using the quasi-steady state approximation in conjunction with our approximated constraints we obtain,

$$\frac{1}{\Gamma}\frac{d}{dt}[\text{pSTAT5}] = \frac{[\text{JAK}_\text{total}][\text{STAT5}]}{[\text{STAT5}](1+\mathcal{I}_J) + k_m(1+\mathcal{I}_J\frac{k_d}{k_m})} - \frac{\lambda}{\Gamma}[\text{pSTAT5}]. \tag{3}$$

Here $\mathcal{I}_J = \frac{k_+^{\text{Ij}}}{k_-^{\text{Ij}}}[\text{I}_\text{jak}]$, $k_d = \frac{k_-}{k_+}$, and lastly $k_m = \frac{\Gamma + k_-}{k_+}$. Equation 3 can be simplified further by considering

$\frac{k_d}{k_m}$ in terms of the rate constants

$$\frac{k_d}{k_m} = \frac{1}{1 + \underbrace{\frac{\Gamma}{k_-}}_{\approx 0}} \approx 1. \tag{4}$$

Incorporating this approximation and noting that Γ sets the time-scale, we change to dimensionless units, $d\tau = \Gamma dt$ and $\Lambda = \frac{\lambda}{\Gamma}$, so that

$$\frac{d}{d\tau}[\text{pSTAT5}] = \frac{[\text{JAK}_{\text{total}}]}{(1+\mathcal{I}_J)} \frac{[\text{STAT5}]}{[\text{STAT5}] + k_m} - \Lambda[\text{pSTAT5}]. \tag{5}$$

In our experimental measurements, we measure pSTAT5 abundance after the system has reached steady state (Supplementary Figure 1). In addition, the measurements are proportional to the abundance of pSTAT5, and not precise concentrations as assumed in our model development. We incorporate these experimental facts by setting the time derivative to zero, introducing a parameter ϕ to appropriately scale our measurements, and rearranging Eq. 5 we obtain,

$$y = \frac{\alpha}{(1+\mathcal{I}_J)} \frac{\phi x - y}{(\phi x - y) + 1}. \tag{6}$$

Here $y = [\text{pSTAT5}]/k_m$ and represents our pSTAT5 measurement, $\phi x = [\text{STAT5}_{\text{total}}]/k_m$ is the adjusted total STAT5 measurement, and $\alpha = \frac{[\text{JAK}_{\text{total}}]}{\Lambda k_m}$ representing the our pSTAT5 amplitude measurements.

To test the mechanism of inhibition we fit our data to the closed form solution

$$\boxed{y = \frac{1}{2}\left[\phi x + 1 + \frac{\alpha}{1+\mathcal{I}_J} - \sqrt{\left(\phi x + 1 + \frac{\alpha}{1+\mathcal{I}_J}\right)^2 - 4\alpha\frac{\phi x}{1+\mathcal{I}_J}}\right]}. \tag{7}$$

Eq. 7 is used to fit our experimental data of the response of pSTAT5 for all levels of total STAT5 and inhibitor dosages. Supplementary Table 1 lists the parameters found to minimize the sum of squared residuals (Supplementary Figure 3).

We then used our model and the inferred parameters to derive new coordinates that transform our data to a straight line with a y-intercept of zero and slope of unity,

$$\underbrace{y_{\text{data}}\left(1+\hat{\mathcal{I}}_J\right)\left(\hat{\phi}x_{\text{data}} - y_{\text{data}} + 1\right)}_{\text{Normalized pSTAT5}} = \underbrace{\hat{\alpha}(\hat{\phi}x_{\text{data}} - y_{\text{data}})}_{\text{Normalized STAT5}}. \tag{8}$$

We see excellent agreement between our transformed data and the predicted values (Main text Fig. 2D).

Parameter	Interpretation	Replicate 1	Replicate 2	Mean
ϕ	prefactor for total STAT expression	4.13	5.05	4.59
α	normalized amplitude	5.65	5.32	5.49
k_I	Inhibitor dissociation constant	5.33 nM	4.93 nM	5.13 nM

Supplementary Table 1: Parameters obtained from minimizing the sum of squared residuals between our experimental data and Equation 7.

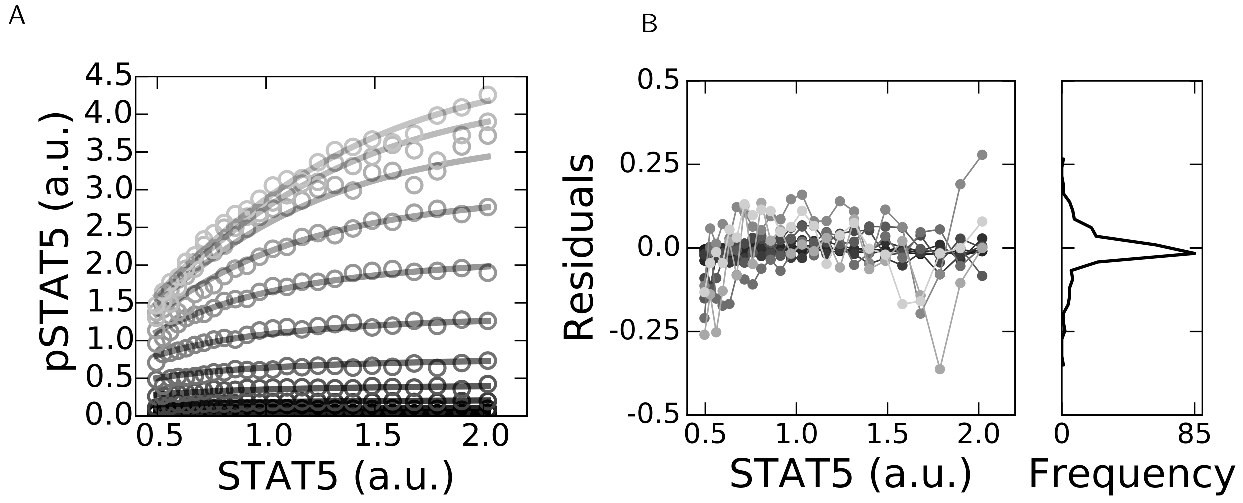

Supplementary Figure 3: (A) The resulting fit of the data (open circles) with the noncompetitive model (lines) presented in Equation 7. (B) The residuals of the data points from the noncompetitive model fit for each dose of JAK inhibitor. The color gradient from gray to black represents increasing AZD1480 concentration, Ij.

Supplementary Note 1.3 Uncompetitive inhibition

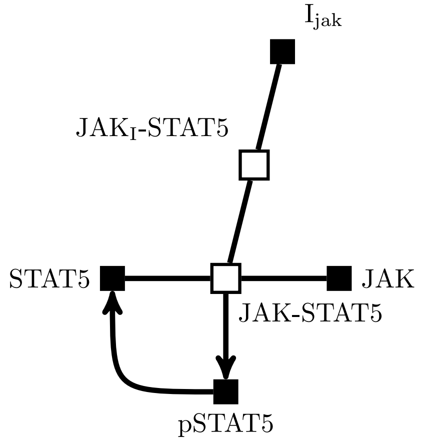

Supplementary Figure 4: Graphical representation of the uncompetitive JAK inhibition model.

The uncompetitive inhibitor is a small molecule that binds exclusively to its target enzyme bound to its cognate substrate - in this case the JAK-STAT5 chemical complex. Indeed, this mechanism of inhibition manifests in the equilibrium equations of the drug-enzyme interaction and the conservation equation for $[JAK_{total}]$. Apart from the inhibitor's dynamic equations our analysis remains the same as the noncompeti-

tive scenario,

$$\frac{d}{dt}[\text{pSTAT5}] = \Gamma[\text{JAK-STAT5}] - \lambda[\text{pSTAT5}], \tag{9a}$$

$$\frac{d}{dt}[\text{JAK-STAT5}] = k_+[\text{STAT5}][\text{JAK}] - k_-[\text{JAK-STAT5}] - \Gamma[\text{JAK-STAT5}] + k_-^{\text{Ij}}[\text{JAK}_\text{I}\text{-STAT5}] - k_+^{\text{Ij}}[\text{I}_\text{jak}][\text{JAK-STAT5}], \tag{9b}$$

$$\frac{d}{dt}[\text{JAK}_\text{I}\text{-STAT5}] = k_+^{\text{Ij}}[\text{I}_\text{jak}][\text{JAK-STAT5}] - k_-^{\text{Ij}}[\text{JAK}_\text{I}\text{-STAT5}]. \tag{9c}$$

The following mass conservation constraint follows as:

$$[\text{STAT5}_\text{total}] \approx [\text{pSTAT5}] + [\text{STAT5}], \tag{10a}$$

$$[\text{JAK}_\text{total}] = [\text{JAK}] + [\text{JAK-STAT5}] + [\text{JAK}_\text{I}\text{-STAT5}]. \tag{10b}$$

Then applying our quasi-steady state approximation as before we obtain an expression for the dynamics of pSTAT5,

$$\frac{d}{d\tau}[\text{pSTAT5}] = [\text{JAK}_\text{total}]\frac{[\text{STAT5}]}{[\text{STAT5}](1+\mathcal{I}_J) + k_m} - \Lambda[\text{pSTAT5}]. \tag{11}$$

Where $\mathcal{I}_J = \frac{k_+^{\text{Ij}}[\text{I}_\text{jak}]}{k_-^{\text{Ij}}}$, $k_m = \frac{k_- + \Gamma}{k_+}$, and $[\text{STAT5}] \approx [\text{STAT5}_\text{total}] - [\text{pSTAT5}]$. Solving for the dimensionless variables describe in Equation 6, we obtain

$$y = \frac{\alpha}{1+\mathcal{I}_J}\ \frac{\phi x - y}{\phi x - y + \frac{1}{1+\mathcal{I}_J}}. \tag{12}$$

This representation provides a transparent understanding of the difference between the uncompetitive and uncompetitive inhibitors. Unlike the noncompetitive inhibitor which reduces the response amplitude exclusively, the uncompetitive inhibitor effectively reduces both the response amplitude and the half effective inhibition concentration.

We conclude by presenting the closed form solution used to fit the data (Supplementary Figure 5A)

$$\boxed{y = \frac{1}{2}\left[\phi x + \frac{1+\alpha}{1+\mathcal{I}_J} - \sqrt{\left(\phi x + \frac{1+\alpha}{1+\mathcal{I}_J}\right)^2 - 4\alpha\frac{\phi x}{1+\mathcal{I}_J}}\right]}. \tag{13}$$

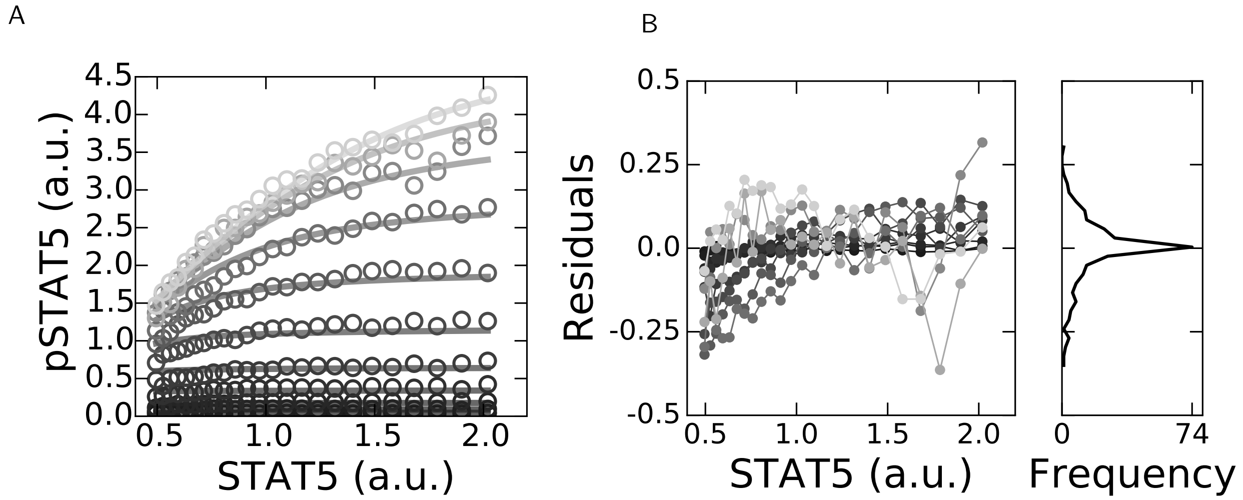

Supplementary Figure 5: (A) The resulting fit of the data (open circles) and the uncompetitive model (lines) represented by Equation 13. (B) The residuals of the data points from the uncompetitive model fit for each dose of JAK inhibitor. The color gradient from gray to black represents increasing AZD1480 concentration, Ij.

Supplementary Note 1.4 Competitive inhibition

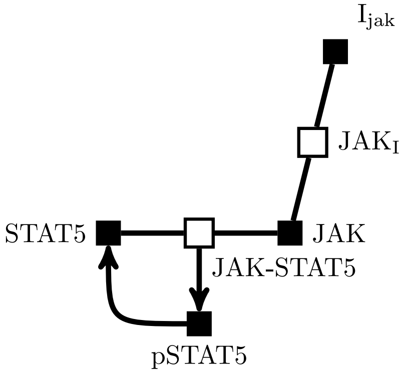

Supplementary Figure 6: Graphical representation of the competive inhibition model.

Competitive inhibitors compete with the substrate for binding to the target enzyme. Apart from the difference in the dynamic equations describing the unique mechanism of action, the proceeding analysis utilizes the same approximations as the previous sections. Incorporating the inhibitor mechanism of action into the dynamics

$$\frac{d}{dt}[\mathrm{pSTAT5}] = \Gamma[\mathrm{JAK\text{-}STAT5}] - \lambda[\mathrm{pSTAT5}], \tag{14a}$$

$$\frac{d}{dt}[\mathrm{JAK\text{-}STAT5}] = k_+[\mathrm{STAT5}][\mathrm{JAK}] - k_-[\mathrm{JAK\text{-}STAT5}] - \Gamma[\mathrm{JAK\text{-}STAT5}], \tag{14b}$$

$$\frac{d}{dt}[\mathrm{JAK_I\text{-}STAT5}] = k_-^{\mathrm{Ij}}[\mathrm{JAK_I}] - k_+^{\mathrm{Ij}}[\mathrm{I_{jak}}][\mathrm{JAK}]. \tag{14c}$$

As before we approximate the mass conservation equations of constraint as

$$[\text{STAT5}_{\text{total}}] \approx [\text{pSTAT5}] + [\text{STAT5}], \tag{15a}$$

$$[\text{JAK}_{\text{total}}] = [\text{JAK}] + [\text{JAK-STAT5}] + [\text{JAK}_{\text{I}}]. \tag{15b}$$

Applying our quasi-steady state approximation

$$\frac{d}{d\tau}[\text{pSTAT5}] = [\text{JAK}_{\text{total}}]\frac{[\text{STAT5}]}{[\text{STAT5}] + k_m(1+\mathcal{I}_J)} - \Lambda[\text{pSTAT5}], \tag{16}$$

with $\mathcal{I}_J = \frac{k_+^{\text{Ij}}[\text{I}_{\text{jak}}]}{k_-^{\text{Ij}}}$, $k_m = \frac{k_- + \Gamma}{k_+}$, and $[\text{STAT5}] = [\text{STAT5}_{\text{total}}] - [\text{pSTAT5}]$. As before we proceed by solving for the unitless variables describe in Eq. 6 and arrive at

$$y = \alpha \frac{\phi x - y}{(\phi x - y) + 1 + \mathcal{I}_J}. \tag{17}$$

From this equation we can identify the unique functional difference between the noncompetitive and uncompetitive inhibitors with the competitive inhibitor. Unlike the former two equations (Eqs 6,12) representing the JAK inhibitor action, the competitive inhibitor increases the half effective concentration of STAT5.

The closed form solution used to fit our data for JAK inhibitor model selection is

$$\boxed{y = \frac{1}{2}\left[\phi x + \alpha + 1 + \mathcal{I}_J - \sqrt{(\phi x + \alpha + 1 + \mathcal{I}_J)^2 - 4\alpha\phi x}\right]}. \tag{18}$$

Supplementary Figure 7A,B, shows the large systematic error when this model is applied to our experimental data.

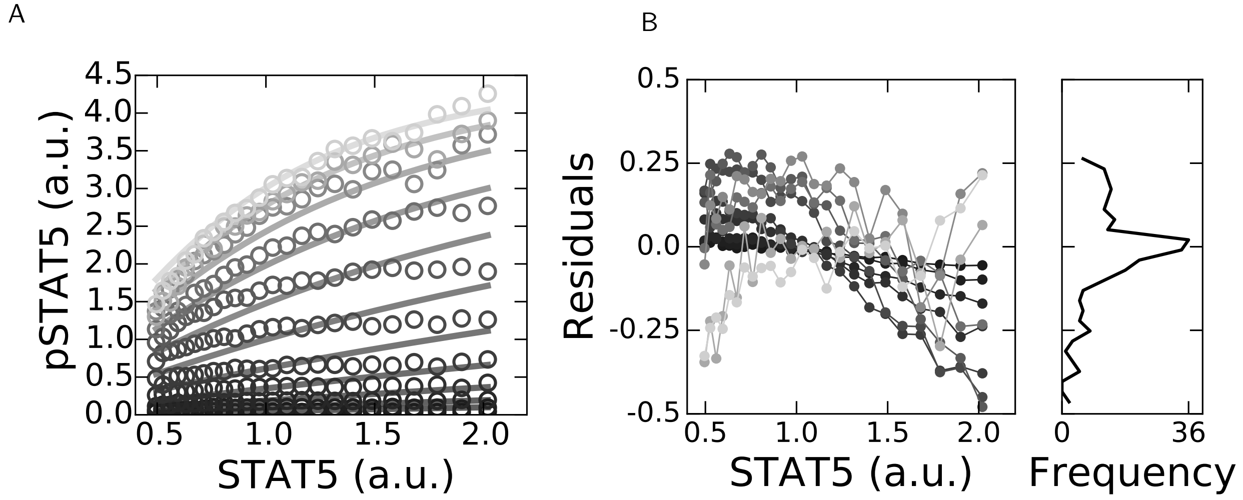

Supplementary Figure 7: (A) The resulting fit of data (open circles) and the competitive inhibitor model model represented in Equation 18 (lines). (B) The residuals of the data points from the uncompetitive model fit for each dose of JAK inhibitor. The color gradient from gray to black represents increasing AZD concentration, Ij.

Supplementary Note 1.5 Model selection

To find the most likely mechanism of action of AZD1480 in living cells we measured the ability of each model to explain our data ($\mathcal{D} = \{([\text{I}_{\text{jak}}], x = \text{STAT5}, y = \text{pSTAT5})\}$). First, we found the optimal parameter set

$(\boldsymbol{\theta}_i)$ for each model, f_i where $i \in$ {Noncompetitive, Uncompetitive, Competitive }, by minimizing the total residuals (ϵ),

$$\hat{\boldsymbol{\theta}}_i = \underset{\boldsymbol{\theta}_i}{\operatorname{argmin}} \left[\epsilon(\boldsymbol{\theta}_i; f_i, \mathcal{D}) \right], \tag{19}$$

where

$$\epsilon(\boldsymbol{\theta}_i; f_i, \mathcal{D}) = \sum_{l=1}^{N} \sum_{m=1}^{M} \left(y_{lm} - f_i \left([\mathrm{I}_{\mathrm{jak}}]_l, x_m; \boldsymbol{\theta}_i \right) \right)^2 \qquad \text{where } \boldsymbol{\theta}_i = \{\alpha_i, \phi_i, k_{Ii}\}. \tag{20}$$

In this equation N is the number of inhibitor doses and M is the number of CCVA determined STAT5 data points. Once $\boldsymbol{\theta}_i$ is found for all i models, we selected the optimal model by,

$$f_{\mathrm{opt}} = \underset{f_i}{\operatorname{argmin}} \left[\epsilon(\hat{\boldsymbol{\theta}}_i; f_i, \mathcal{D}) \right]. \tag{21}$$

In Fig. 2B of the main text we present the total residuals for each model used in Eq. 21.

Other metrics (*e.g.* AIC, BIC) could be used for selecting the optimum model. These techniques are essential for comparing models with different number of parameters. However, no such technique is required for our analysis, because each model has the same number of parameters.

Supplementary Note 2 Coarse grained model of TCR signaling

We developed a coarse-grained model founded upon previous models of T cell receptor (TCR) activation and phosphorylation of ERK [4, 5, 6]. In our model we simplify the network to five components: 1) peptide bound MHC and TCR complex which we designate "$L-R$", 2) the SRC family kinase Lck, which is bound the the intracellular domain of CD8, designated "SRC", 3) the active receptor complex as "SRC*", 4) phosphorylated MEK designated "pMEK", and 5) phosphorylated ERK designated "ppERK". The variables and their interactions with one another are represented in Supplementary Figure 8.

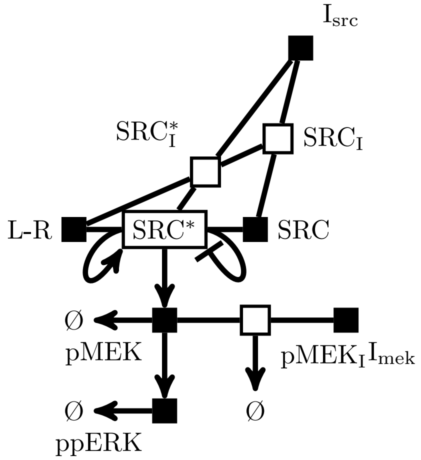

Supplementary Figure 8: Coarse-grained TCR signaling model. Empty squares designate chemical complexes, while filled squares are independent chemical species. Arrow-less lines connecting two squares indicates an equilibrium reaction, while lines with arrows indicate enzymatic reactions.

The components interact according the following mass action differential equations:

$$\begin{aligned}\frac{d}{dt}[\mathrm{SRC}^*] = k_+[L-R][\mathrm{SRC}] - k_-[\mathrm{SRC}^*] + \\ k_+[L-R][\mathrm{SRC}]\frac{\Gamma_+[\mathrm{SRC}^*]^n}{[\mathrm{SRC}^*]^n + k_{m+}^n} - k_-\frac{\Gamma_-[\mathrm{SRC}^*]}{[\mathrm{SRC}^*]+k_{m-}} + \\ k_-^{\mathrm{Is}}[\mathrm{SRC}_\mathrm{I}^*] - k_+^{\mathrm{Is}}[\mathrm{SRC}^*][I_{\mathrm{SRC}}],\end{aligned} \tag{22a}$$

$$\frac{d}{dt}[\mathrm{SRC_I}] = k_+^{\mathrm{Is}}[\mathrm{SRC}][I_{\mathrm{SRC}}] - k_-^{\mathrm{Is}}[\mathrm{SRC_I}] + k_-[\mathrm{SRC}_\mathrm{I}^*] - k_+[\mathrm{SRC_I}][L-R], \tag{22b}$$

$$\frac{d}{dt}[\mathrm{pMEK_{total}}] = \Gamma_M\frac{[\mathrm{SRC}^*]^N}{[\mathrm{SRC}^*]^N + k^N} - \lambda[\mathrm{pMEK_{total}}], \tag{22c}$$

$$\frac{d}{dt}[\mathrm{pMEK_I}] = k_+^{\mathrm{Im}}[\mathrm{pMEK}][I_{\mathrm{MEK}}] - k_-^{\mathrm{Im}}[\mathrm{pMEK_I}], \tag{22d}$$

$$\frac{d}{dt}[\mathrm{ppERK}] = \Gamma_E[\mathrm{pMEK}] - \lambda[\mathrm{ppERK}]. \tag{22e}$$

In this model all equilibrium rate parameters are represented using $k^{\theta}_{+,-}$ notation, where $(+,-)$ represent the forward and reverse rates, respectively, and θ provides the corresponding reaction identity. Specifically, no superscript represents the $L-R$ and SRC interaction, 'Is' superscript is the SRC and I_{SRC} interaction, and lastly 'Im' superscript is the pMEK and I_{MEK} interaction. The feedbacks are parameterized by the half effective concentration constants for the forward reaction k_{m+} and the reverse reaction k_{m-}, the hill coefficient of the positive feedback n, and the positive (Γ_+) and negative (Γ_-) feedback strengths. Lastly, all dephosphorylation rates, λ, are identical.

Analysis of the equations are broken into two portions - first the dynamics of [SRC*], followed by those of [pMEK] and [ppERK]. To analyze the dynamics of [SRC*] we first define the constraints originating from

mass conservation as

$$[\mathrm{SRC_{total}}] = [\mathrm{SRC}] + [\mathrm{SRC}^*] + [\mathrm{SRC_I}] + [\mathrm{SRC_I^*}], \tag{23a}$$

$$[L - R_{\mathrm{total}}] = [L - R] + [\mathrm{SRC}^*] + [\mathrm{SRC_I^*}]. \tag{23b}$$

In our experiments we administer saturating doses of antigen bound to peptide MHC on antigen presenting cells, $[L - R_{\mathrm{total}}] \gg [\mathrm{SRC_{total}}]$, making $[L - R_{\mathrm{total}}] \approx [L - R]$ a reasonable approximation. Furthermore, we apply a quasi-steady state approximation in which all equilibrium reactions are much faster than enzymatic reactions. Together we see that

$$[\mathrm{SRC}] = \frac{[\mathrm{SRC_{total}}]}{1 + \frac{[I_{\mathrm{SRC}}]}{k_I^{\mathrm{src}}}} - [\mathrm{SRC}^*] \tag{24}$$

with $k_I^{\mathrm{src}} = k_-^{\mathrm{Is}}/k_+^{\mathrm{Is}}$.

We continue by normalizing our data by k_{m+} allowing us to rewrite the dynamics of $[\mathrm{SRC}^*]$ by the unitless variable x. Specifically we take $x = [\mathrm{SRC}^*]/k_{m+}$, $\alpha = k_{m-}/k_{m+}$, $s_t = [\mathrm{SRC_{total}}]/k_{m+}$, $\Gamma_-/k_{m+} = \Gamma'_-$, and $\mathcal{I}_s = I/k_I^{\mathrm{src}}$ to obtain

$$\frac{1}{\Gamma'_- k_-}\frac{d}{dt}x = \frac{\Gamma_+[L-R]}{\Gamma'_- k_d}\left[\frac{1}{\Gamma_+} + \frac{x^n}{x^n+1}\right]\left[\frac{s_t}{1+\mathcal{I}_s} - x\right] - \left[\frac{1}{\Gamma'_-} + \frac{1}{x+\alpha}\right]x. \tag{25}$$

Throughout our experiments we do not change the quantity of antigen or antigen presenting cells allowing us to group $\frac{\Gamma_+[L-R]}{\Gamma_- k_d}$ into a single dimensionless parameter A_1. Furthermore, we take the feedback amplitudes to be much larger than 1, resulting in $\frac{1}{\Gamma_+}, \frac{1}{\Gamma'_-} \to 0$ and a simpler expression for x,

$$\frac{d}{d\tau}x \approx A_1 \frac{x^n}{x^n+1}\left[\frac{s_t}{1+\mathcal{I}_s} - x\right] - \frac{x}{x+\alpha}. \tag{26}$$

This simplified equation of the dimensionless variable x provides a function for analyzing the topology dependent dynamic phenotype of our system. To find the values of x at the fixed points of the system we numerically evaluated the stationary solution to Equation 26.

The second component of our system represents the dynamics of [pMEK] and [ppERK]. The MEK inhibitor binds to free pMEK, which results in an inactive complex pMEK-I_{MEK}. As a result the total pMEK ($\mathrm{pMEK_{total}}$) produced by SRC^* exists in two states. To link the dynamic equation governing $[\mathrm{pMEK_{total}}]$ (Eq 22c) with the uninhibited pMEK species - the species that produces our quantity of interest ([ppERK]) - we write the following mass conservation equation $[\mathrm{pMEK_{total}}] = [\mathrm{pMEK}] + [\mathrm{pMEK_I}]$. In addition, we assume that the kinetics of the drug binding to MEK are much faster than the enzymatic reactions. Incorporating the constraint and approximation we see that $[\mathrm{pMEK_{total}}] = [\mathrm{pMEK}](1 + k_I^{\mathrm{mek}})$, allowing us to proceed by writing an expression for the dynamics of [pMEK] with respect to Eq 22c as

$$\frac{d}{dt}[\mathrm{pMEK}] = \frac{\Gamma_M}{1+\mathcal{I}_m}\frac{[\mathrm{SRC}^*]^N}{[\mathrm{SRC}^*]^N + k^N} - \lambda[\mathrm{pMEK}]. \tag{27}$$

We continue by rewriting the expression with respect to dimensionless variables and obtain

$$\frac{d}{d\tau}m = \frac{\gamma_m}{1+\mathcal{I}_m}\frac{x^N}{x^N + k_x^N} - \Lambda m. \tag{28}$$

Where $\gamma_m = \frac{\Gamma_M}{k_- \Gamma_-}$, $\Lambda = \frac{\lambda k_{m+}}{k_- \Gamma_-}$, $x = [\mathrm{SRC}^*]/k_{m+}$, $k_x = k/k_{m+}$, and $m = [\mathrm{pMEK}]/k_{m+}$. Similarly, we rearrange the dynamics of [ppERK] to obtain

$$\frac{d}{d\tau}y = \gamma_e m(\tau) - \Lambda y, \tag{29}$$

where $\gamma_e = \frac{\Gamma_e k_{m+}}{k_- \Gamma_-}$ and $y = [\mathrm{ppERK}]/k_{m+}$.

By solving the differential equation for m we can simplify our system of three equations to two. Two simple dynamic equations is ideal, because the system properties can be easily interpreted in a single two-dimensional phase diagram (Figures 4 and 5 in the main text). The m equation can be described as an integral equation

$$m(\tau) = \frac{\gamma_m}{\Lambda}\frac{1}{1+\mathcal{I}_s}\left[\underbrace{\frac{x^N(\tau_o)}{x^N(\tau_o)+k_x^N}e^{-\Lambda(\tau-\tau_o)}}_{A} + \underbrace{\Lambda\int_{s>\tau_o}^{\tau}\frac{x^N(s)}{x^N(s)+k_x^N}e^{-\Lambda(\tau-s)}ds}_{B}\right]. \tag{30}$$

The integral equation representation provides transparency to the systems behavior at the initial conditions, $\tau = \tau_o$, designated A, and the resulting dynamics, $\tau > \tau_o$, designated B. This construction is useful to understanding the stream plots in Figure 4 of the main text. In this figure we propose a hypothetical experiment. In this experiment we imagine that it is possible to perfectly manipulate the system, which results in our ability to place it at any coordinate pair (x, m, y). Our question then is, what will be the initial velocity of our system be if we allow it to evolve in accordance to its nature. Mathematically, this hypothetical experiment results in the exclusive evaluation of $\tau = \tau_o$, which results in our setting $B = 0$. Substituting for m and $\gamma_m\gamma_e/\Lambda^2 = A_0$, the initial fluxes are described as

$$\left.\frac{d}{d\tau}x(\tau)\right|_{\tau=\tau_o} = A_1\frac{x(\tau_o)^n}{x(\tau_o)^n+1}\left[\frac{s_t}{1+\mathcal{I}_s} - x(\tau_o)\right] - \frac{x(\tau_o)}{x(\tau_o)+\alpha}, \tag{31a}$$

$$\left.\frac{d}{d\tau}y(\tau)\right|_{\tau=\tau_o} = \frac{\Lambda A_0}{1+\mathcal{I}_m}\frac{x(\tau_o)^N}{x(\tau_o)^N+k_x^N} - \Lambda y(\tau_o). \tag{31b}$$

The Equations 31 represent the instantaneous flux and are used for the stream plots of the [ppERK] versus [SRC*] (y and x) phase planes in the main text. We proceed by finding the properties of our equations that are experimentally measured - namely the locations and the respective stabilities of the stationary values,

$$0 = A_1\frac{x^n}{x^n+1}\left[\frac{s_t}{1+\mathcal{I}_s} - x\right] - \frac{x}{x+\alpha}, \tag{32a}$$

$$y = \frac{A_0}{1+\mathcal{I}_m}\frac{x^N}{x^N+k_x^N}. \tag{32b}$$

We evaluated the fixed points of x and their corresponding stability numerically in accordance to Eq 32a. The stability of each fixed point was assessed by numerical evaluating the direction of the flux, positive or negative, for small displacements about the respective fixed point. Given our chosen parameter set (Supplementary Table 2), we found our system to elicit bistable behavior [7].

Parameter	Interpretation	Assignment
A_1	Effective x forward rate constant	1.75
r_t	Quantity of dimensionless [SRC_{total}]	2.5
n	steepness of positive feedback	3
α	negative feedback sensitivity / positive feedback sensitivity	0.1
A_0	Effective y production constant	1
k_x	sensitivity of y to changes in x	0.5
N	hill coefficient of MAPK	4.
Λ	Effective y degradation constant	1.

Supplementary Table 2: Model parameters used for the main text figures.

Supplementary Note 3 Single cell signaling data

In this section we present our single cell signaling data of TCR signaling response to various SRC and MEK inhibitors. We use measurements of MEK(pS221), *a.k.a.* pMEK, and ERK(pT303pY204), *a.k.a.* ppERK, abundances per cell to quantify the signaling pathway's response to small-drug inhibition.

Supplementary Note 3.1 SRC inhibitors

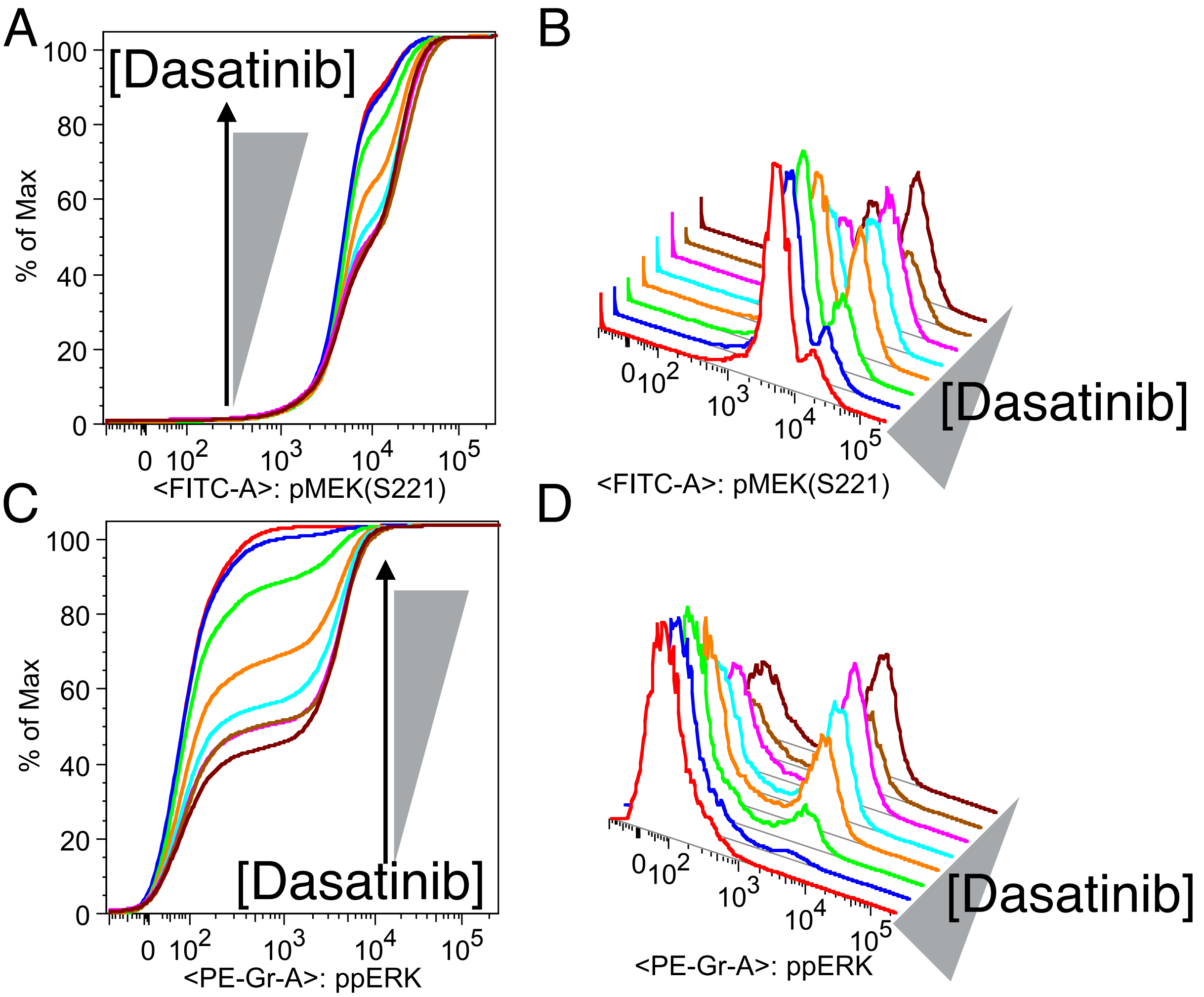

Supplementary Figure 9: **Single cell response to SRC inhibitor Dasatinib.** These data are also used in the main text of the manuscript. We present the pMEK (pS221) and ppERK response for 8 unique doses obtained from a 1.5 fold serial dilution starting with 1 μM Dasatinib. (A) The cumulative frequency of single cell pMEK abundance in response to Dasatinib. (B) Histograms of pMEK response for the identical doses of Dasatinib as presented in 'A'. (C) The cumulative frequencies and (D) histograms of ppERK abundance per cell for identical doses of Dasatinib as presented in 'A' and 'B'.

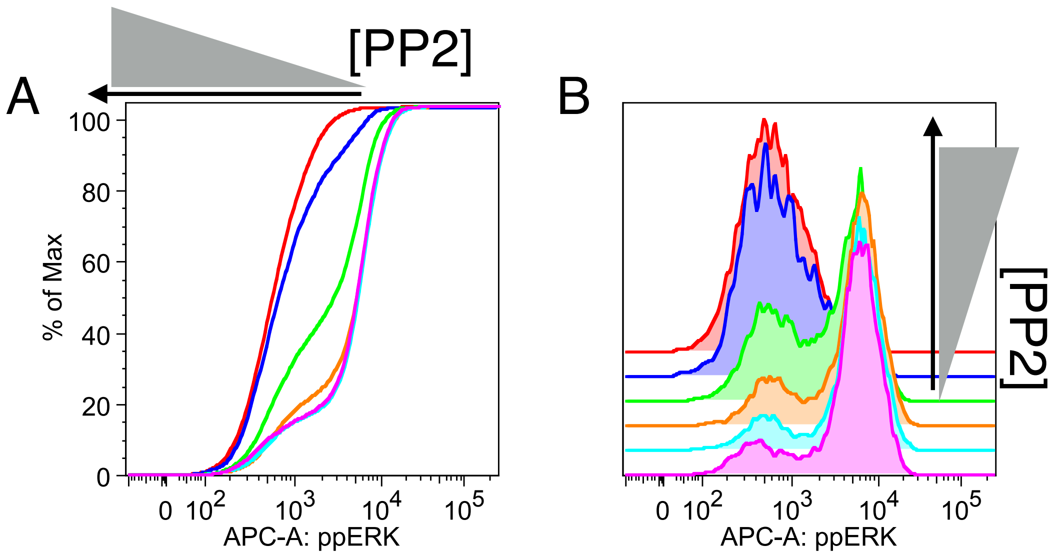

Supplementary Figure 10: **Single cell response to SRC inhibitor PP2.** (A) Cumulative distribution and (B) histograms of single cell ppERK response to 2 fold serial dilution of SRC inhibitor PP2 (starting concentration is 50 μMol).

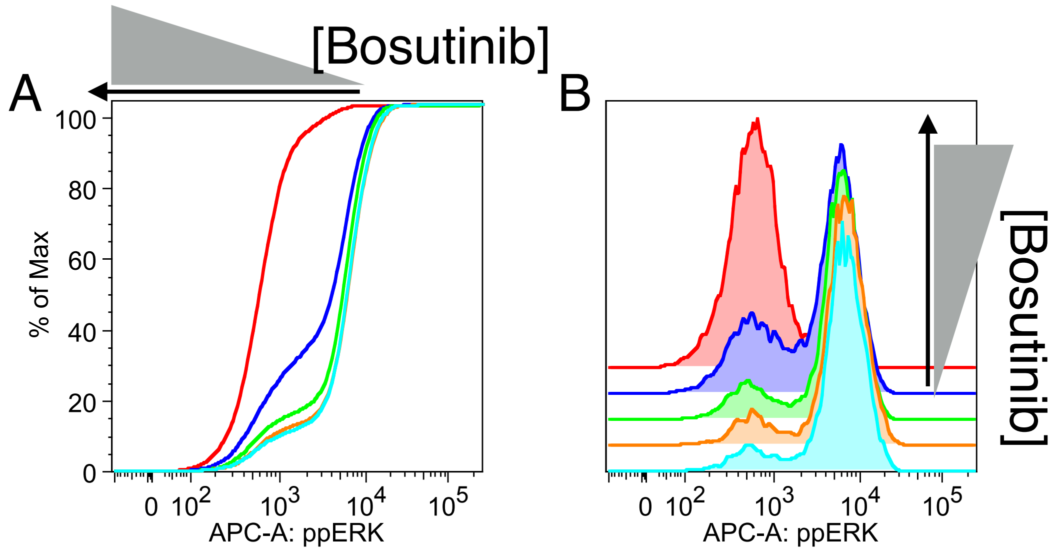

Supplementary Figure 11: **Single cell response to SRC inhibitor Bosutinib.** (A) Cumulative distribution and (B) histograms of single cell ppERK response to 2 fold serial dilution of SRC inhibitor Bosutinib (starting concentration is 1 μMol).

Supplementary Note 3.2 MEK Inhibitors

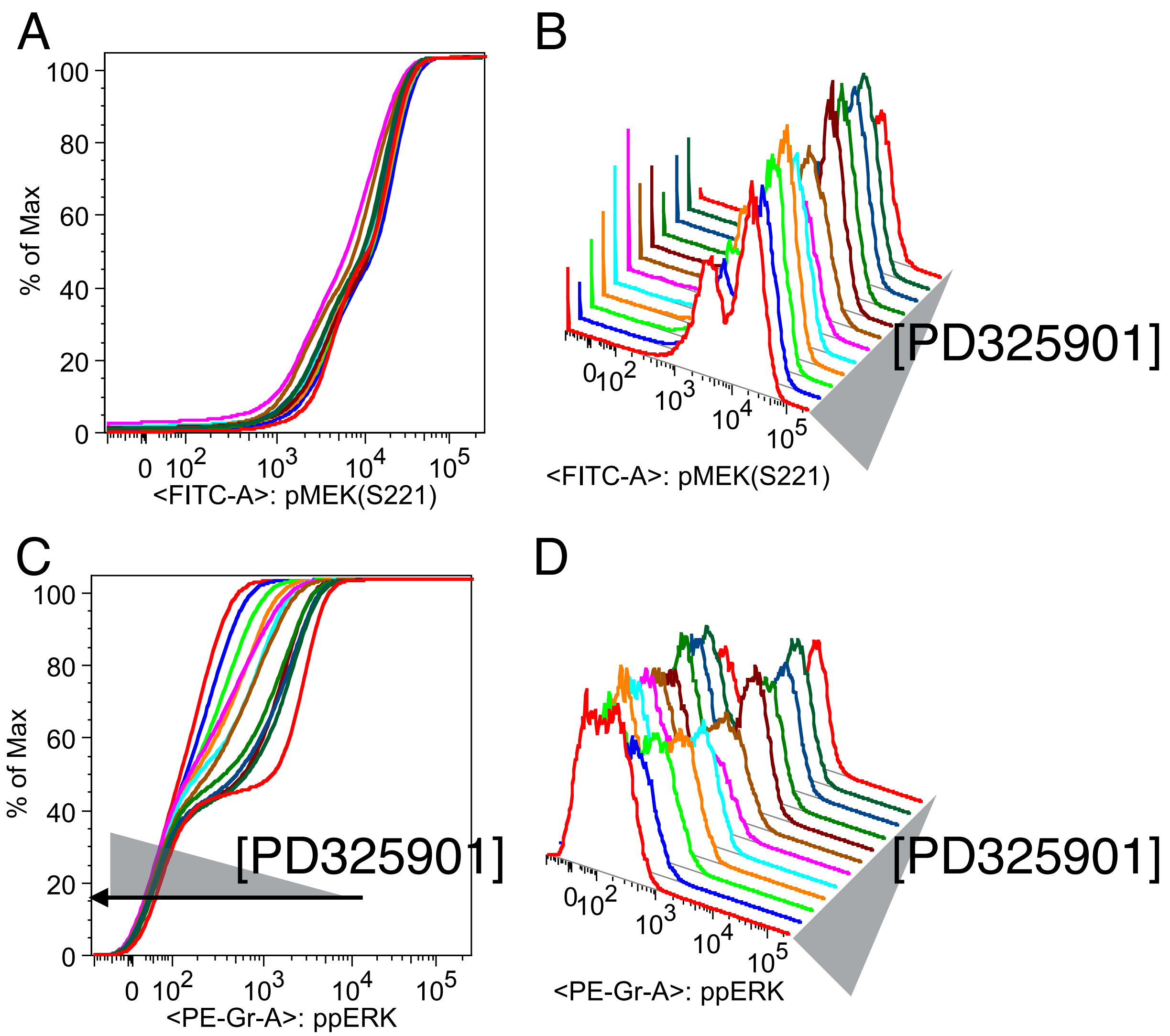

Supplementary Figure 12: **Single cell response to MEK inhibitor PD325901.** These data are also used in the main text of the manuscript. We present the pMEK (pS221) and ppERK response for 12 unique doses obtained from a 2 fold serial dilution starting with 1 μM PD325901. (A) The cumulative frequency of single cell pMEK abundance in response to PD325901. (B) Histograms of pMEK response for the identical doses of PD325901 as presented in 'A'. (C) The cumulative frequencies and (D) histograms of ppERK abundance per cell for identical doses of PD325901 as presented in 'A' and 'B'.

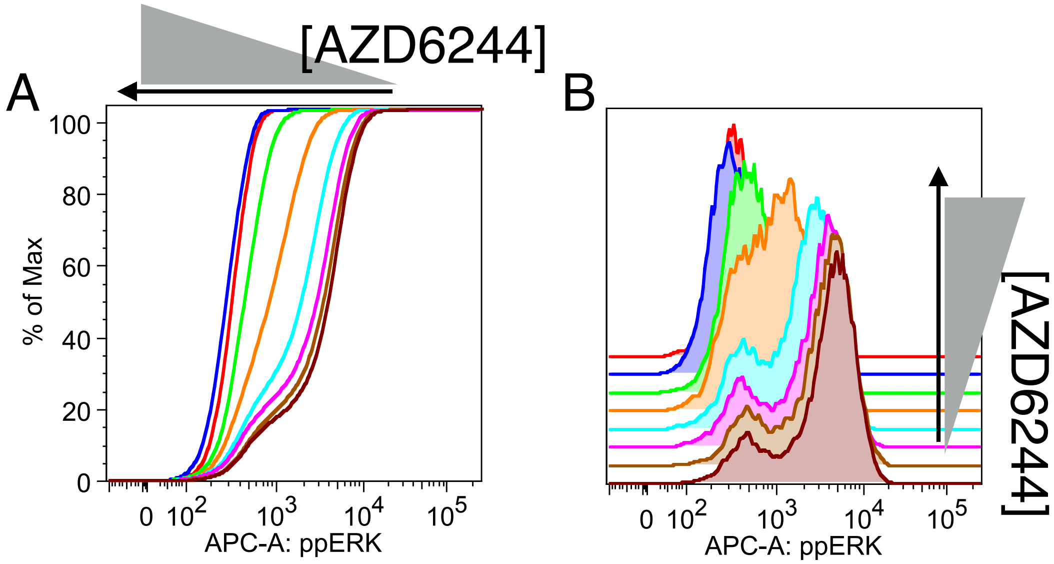

Supplementary Figure 13: **Single cell response to MEK inhibitor AZD6244.** (A) Cumulative distribution and (B) histograms of single cell ppERK response to 2 fold serial dilution of MEK inhibitor AZD6244 (starting concentration is 5 μMol).

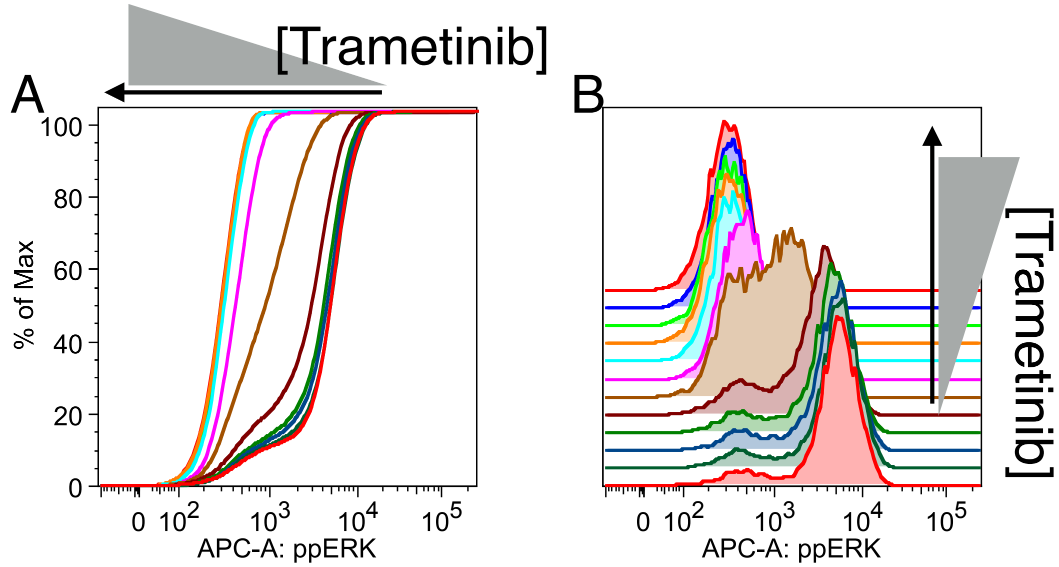

Supplementary Figure 14: **Single cell response to MEK inhibitor Trametinib.** (A) Cumulative distribution and (B) histograms of single cell ppERK response to 2 fold serial dilution of MEK inhibitor Trametinib (starting concentration is 1 μMol).

Supplementary Note 3.3 pMEK (S221) response to MEKi

To ensure the model is correct, and that MAPK is truly a separate and unidirectional sub-network, we measured the response of phosphorylated MEK (pS221; monoclonal antibody (166F8) from Cell Signaling Technologies) to MEK inhibition by PD325901 (Supplementary Figure 12). We quantified the data in accordance with the analysis of ppERK in the main text. Specifically we applied a two component Gaussian mixture model to quantify the means of the pMEK high and pMEK low components and the fraction of cells occupying the pMEK high state. In Supplementary Figures 15 and 16, we show that pMEK abundance and the fraction of cells occupying the high, and consequently the low, states are invariant to MEK inhibition by PD325901.

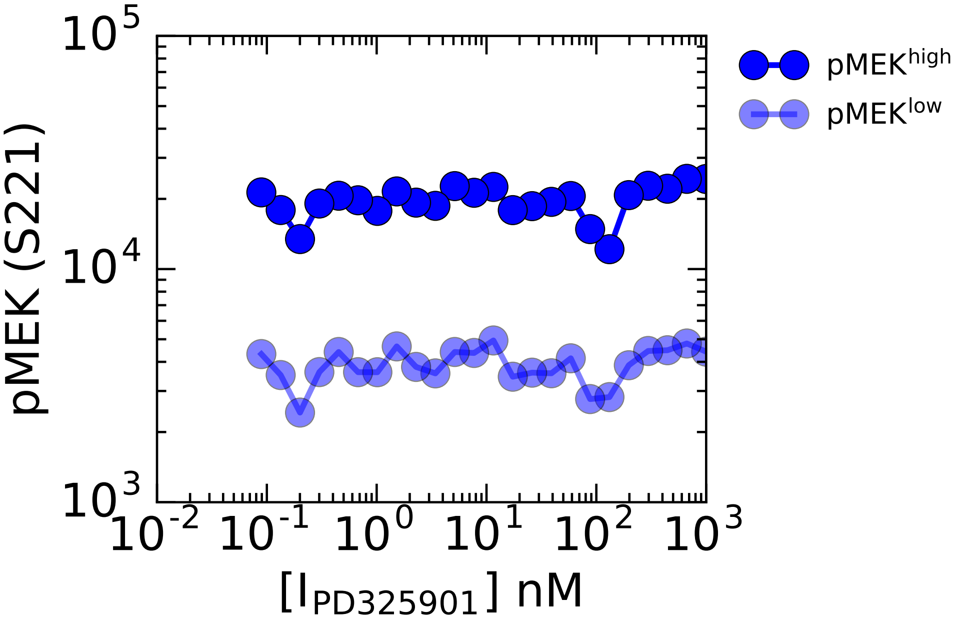

Supplementary Figure 15: **The average of pMEK signal is invariant to MEK inhibition.** The means of each Gaussian mixture component for different doses of MEK inhibitor PD325901.

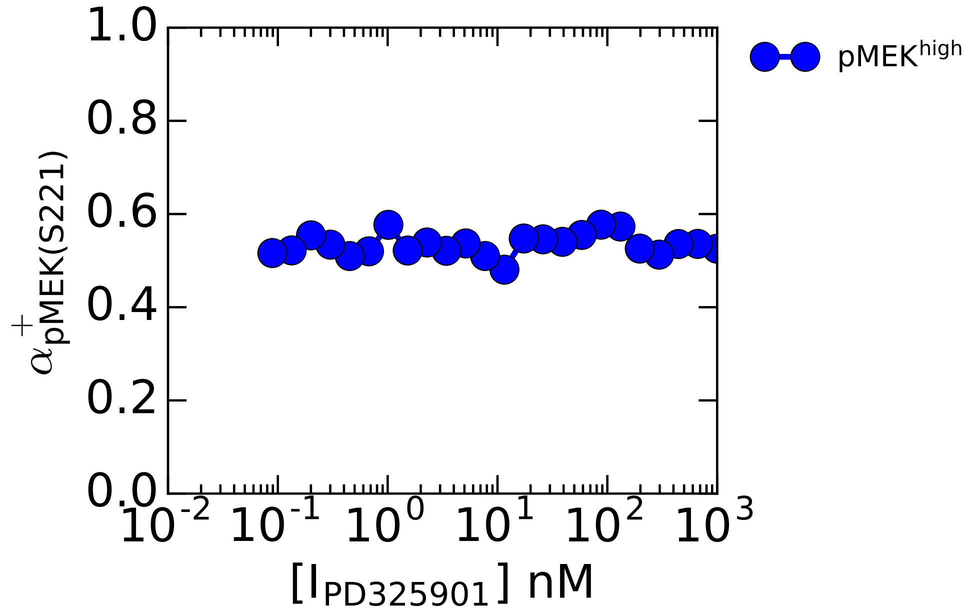

Supplementary Figure 16: **Fraction of cells with pMEK high signal is invariant to MEK inhibition.** The fraction of cells occupying the $pMEK^{high}$ Gaussian mixture component does not change with dose of the MEK inhibitor PD325901

Supplementary Note 4 Proliferation

We extend our signaling findings to the proliferative response of cells to MEK and SRC inhibition by analyzing the dilution of amine reactive dyes (CFSE or CTV), cell size, and the abundance of CD8 per cell. Analogous to the binary activation of ppERK signaling, we classified individual cells as either inactive or active by using a binary gate that separated small and $CD8^{low}$ expressing cells from large and $CD8^{high}$ expressing cells, respectively. To correlate a reduction in the average abundance of ppERK to a functional decrease in proliferation, we computed a normalized average number of divisions among active cells: every time a cell divides from generation n to $n+1$, it contributes an additional sister to the count of $n+1$, and this doubling in cell number must be taken into account when computing the average number of divisions a cell has undergone. Hence, we introduced two observables, the fraction of activated cells and the average number of divisions $\langle \text{Divisions} \rangle$.

We computed the normalized average number of divisions as:

$$\langle \text{Divisions} \rangle = \frac{1}{N_{total}} \sum_{n=0}^{n_{max}} \frac{N_n}{2^n} n, \tag{33}$$

with $N_{total} = \sum_{n=0}^{n_{max}} N_n/2^n$. In this expression the 2^n accounts for the contribution of sister cells for the nth division. We then computed the fraction of active cells and noticed that the effectiveness of our binary gate ($CD8^{low}$ vs $CD8^{high}$) did not report accurately the dynamics of cell proliferation and drug inhibition. Specifically some of the cells gated as inactive (*i.e.* $CD8^{low}$ cells are capable of diluting the CTV dye and as a result should have been counted as active (cells cannot divide unless they have been activated). To accurately count these dividing cells as active, we devised a strategy for the robust estimation of the fraction active by computing the average divisions of the active fraction, $\left\langle \text{Divisions}^{+} \right\rangle$, and the average divisions over all cells $\left\langle \text{Divisions}^{all} \right\rangle$. From the ratio of average divisions,

$$\text{Robust Fraction Activated} = \frac{\left\langle \text{Divisions}^{all} \right\rangle}{\left\langle \text{Divisions}^{+} \right\rangle}, \tag{34}$$

$$= \frac{N_{total}^{+}}{N_{total}^{all}} \frac{\sum_n \frac{N_n^{all}}{2^n} n}{\sum_n \frac{N_n^{+}}{2^n} n}, \tag{35}$$

$$= \text{Fraction Active} \underbrace{\frac{\sum_n n \frac{N_n^{all}}{2^n}}{\sum_n n \frac{N_n^{+}}{2^n}}}_{\text{Gate correction}}, \tag{36}$$

we derived a correction for the gating strategy. Ultimately, we reported $\left\langle \text{Divisions}^{\text{active}} \right\rangle$ and the Robust Fraction Activated in Figure 6C of the main text: these observables best encapsulated the dynamics of lymphocyte response in our system.

Supplementary References

[1] Lineweaver, H. & Burk, D. The Determination of Enzyme Dissociation Constants. *Journal of the American Chemical Society* **56**, 658-666 (1934).

[2] Cornish-Bowden, A. A Simple Method for Determining the Inhibition Constants of Mixed, Uncompetitive and Non-Competitive Inhibitors. *Biochem. J.* **137**, 143-144 (1974).

[3] Feinerman, O. *et al.* Single-cell quantification of IL-2 response by effector and regulatory T cells reveals critical plasticity in immune response. *Molecular Systems Biology* **6**, 437 doi:10.1038/msb.2010.90 (2010).

[4] Altan-Bonnet, G. & Germain, R. N. Modeling T Cell Antigen Discrimination Based on Feedback Control of Digital ERK Responses. *PLoS Biol* **3**, e356 doi:10.1371/journal.pbio.0030356 (2005).

[5] Feinerman, O., Veiga, J., Dorfman, J. R., Germain, R. N. & Altan-Bonnet, G. Variability and Robustness in T Cell Activation from Regulated Heterogeneity in Protein Levels. *Science* **321**, 1081-1084 (2008).

[6] François, P., Voisinne, G., Siggia, E. D., Altan-Bonnet, G. & Vergassola, M. Phenotypic model for early T-cell activation displaying sensitivity, specificity, and antagonism. *Proceedings of the National Academy of Sciences* **110**, E888-E897 (2013).

[7] Strogatz, S. H. *Nonlinear Dynamics and Chaos with applications to Physics, Biology, Chemistry, and Engineering* (Westview Press, USA, MA, Cambridge, 2000).
